# PIK3CA-related overgrowth spectrum (PROS) presenting as isolated macrodactyly

**DOI:** 10.1093/jscr/rjad549

**Published:** 2023-10-14

**Authors:** Kritika Krishnamurthy, Ukuemi Edema, Berrin Ustun, Esperanza Villanueva-Siles, Steven M Koehler, Rizwan Naeem, Yanhua Wang, Doctor Y Goldstein

**Affiliations:** Department of Pathology, Montefiore Medical Center, Bronx, New York 10467, United States; Department of Pathology, Montefiore Medical Center, Bronx, New York 10467, United States; Department of Pathology, Montefiore Medical Center, Bronx, New York 10467, United States; Albert Einstein College of Medicine, Department of Pathology, Bronx, New York 10461, United States; Department of Pathology, Montefiore Medical Center, Bronx, New York 10467, United States; Albert Einstein College of Medicine, Department of Pathology, Bronx, New York 10461, United States; Albert Einstein College of Medicine, Department of Pathology, Bronx, New York 10461, United States; Department of Orthopedic Surgery, Montefiore Medical Center, Bronx, New York 10467, United States; Department of Pathology, Montefiore Medical Center, Bronx, New York 10467, United States; Albert Einstein College of Medicine, Department of Pathology, Bronx, New York 10461, United States; Department of Pathology, Montefiore Medical Center, Bronx, New York 10467, United States; Albert Einstein College of Medicine, Department of Pathology, Bronx, New York 10461, United States; Department of Pathology, Montefiore Medical Center, Bronx, New York 10467, United States; Albert Einstein College of Medicine, Department of Pathology, Bronx, New York 10461, United States

**Keywords:** PIK3CA-related overgrowth spectrum, PROS, macrodactyly, PIK3CA, mTOR pathway, mTOR inhibitors

## Abstract

PIK3CA-related overgrowth spectrum (PROS) is a heterogeneous group of diseases, with varied clinical presentations ranging from isolated segmental overgrowths to megalencephaly and vascular malformations, all resulting from post-zygotic activating mutations in PIK3CA. Isolated macrodactyly of upper limb is extremely rare, accounting only for 0.9%–1% of all congenital anomalies of the upper limb. This report describes a case of congenital, isolated, nonprogressive macrodactyly of the right index finger and thumb, in an adult patient that was treated with debulking surgery. The microscopic features were compatible with lipomatosis of nerve. Due to the prompt and pertinent molecular testing, which identified a somatic PIK3CA variant, c.3140A > G, p.H1047R., the case was classified as a PROS. The availability of mTOR inhibitors offers additional treatment possibilities in cases with progressive disease. This case report highlights the importance of molecular testing to identify PROS, to further the knowledge of this continually expanding entity.

## Introduction

PIK3CA-related overgrowth spectrum (PROS) is a heterogeneous group of diseases, classified under rare genetic developmental defect during embryogenesis [1, 2]. PROS have varied clinical presentations ranging from isolated segmental overgrowths to megalencephaly and vascular malformations, all resulting from post-zygotic activating mutations in PIK3CA, the gene encoding the catalytic alpha subunit of phosphatidylinositol-4,5-bisphosphate 3-kinase (PI3K) [3, 4]. The wide and diverse clinical spectrum is attributed to the variation in timeline of the somatic mutation that can occur at any point in the embryonic development [5]. Isolated macrodactyly of upper limb is extremely rare, accounting only for 0.9%–1% of all congenital anomalies of the upper limb [6]. Unilateral macrodactyly of fingers is classified as a rare subtype of PROS (ORPHANET:295239). This case report highlights the importance of vigilant investigation of isolated macrodactyly, to identify this rare subtype of PROS.

## Case report

A 25-year-old African–American transgender female presented to congenital hand surgery clinic with a congenital enlargement of right index finger and thumb. She denied any numbness, tingling, or altered sensations over the area of deformity. The expansion was static/proportional. Apart from her gender affirming procedure, she did not have any significant past medical or surgical history.

On focused physical examination of her right hand, extensive fatty enlargement of the ulnar thumb and radial index finger, extending into the first web space were seen. There was accompanying dorsal hand enlargement as well. The flexion range was diminished in both the involved digits, but sensation to light touch was intact. The left hand and other extremities were normal in appearance, range of motion, and sensations.

Non-contrast magnetic resonance imaging of the right hand revealed prominence of the subcutaneous fat surrounding the thumb, within the first webspace and radial aspect of the index finger with enlargement of the median nerve branches within this area of prominent subcutaneous fat. The radiological impression was a median nerve lipomatosis (macrodystrophia lipomatosa) with associated nerve territory overgrowth of the soft tissues in the thumb, first webspace, and radial aspects of the index finger.

**Figure 1:**
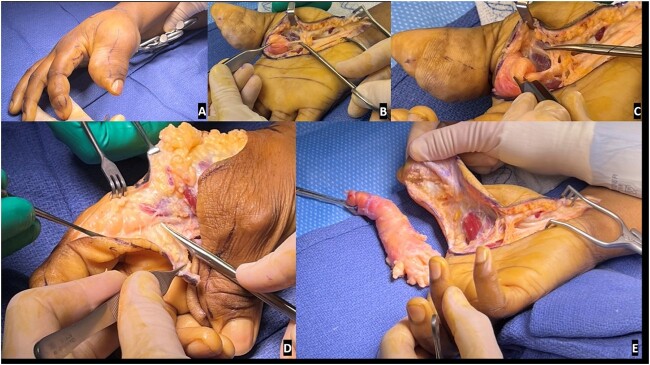
Preoperative and intraoperative images of the macrodactyly: (A) preoperative pictures demonstrating the isolated macrodactyly of the thumb and index finger; (B) scissor is outlining the normal digital nerve to the thumb, which then expands into a lipofibromatous hamartoma (LFH) outlined by the Adson forceps; (C) zoom image demonstrating the difference in the normal radial digital nerve to the thumb and the LFH involved ulnar digital nerve to the thumb; (D) similar image of the normal radial digital nerve to the index finger, expanding into the LFH tumor driving the macrodactyly as noted by the scissors; (E) resection of the LFH tumor of the ulnar digital nerve of the thumb and associated debulking.

Extensive debulking of the lipofibromatous hamartoma involving nerves, subcutaneous tissue, and skin was performed and the debrided tissue was sent to pathology (Fig. 1). Notably, a complete debulking was not feasible, as the thumb contained circumferential aspects of enlargement and debulking would have resulted in potential vascular embarrassment and necrosis.

**Figure 2:**
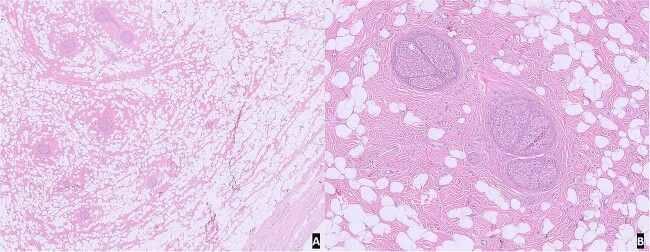
H&E slide from representative sections: (A) low power view of unremarkable skin and subcutaneous tissue with benign nerve branches showing expansion of epineurium; (B) high power view showing expansion of epineurium by adipose tissue and fibrous tissue.

On gross examination, the specimen was composed of multiple fragments of yellow lobular adipose tissue, tan-pink soft tissue, and skin. No hemorrhage, necrosis, or discrete masses were identified. Microscopic examination of H&E slides from representative sections revealed unremarkable skin and subcutaneous tissue with benign nerve branches showing expansion of epineurium by adipose tissue and fibrous tissue. The morphological features were compatible with lipomatosis of nerve (Fig. 2).

**Figure 3:**
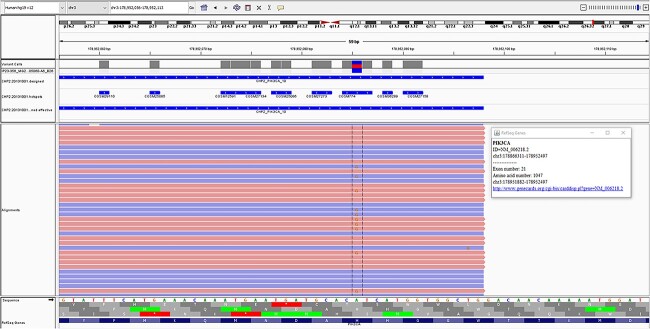
PIK3CA variant, c.3140A > G, p.H1047R, identified in the case, as seen on the integrated genomics viewer.

The formalin-fixed paraffine-embedded tissue was sent to the molecular pathology lab where sequencing was performed using the Ion AmpliSeq™ Cancer Hotspot Panel v2 on The Ion TorrentT™ Personal Genome Machine™ (PGM) system (ThermoFisher Scientific, CA, USA). The panel tests for 97 known pathogenic variants spread across 11 different amplicons of PIK3CA. A somatic PIK3CA variant, c.3140A > G, p.H1047R, was identified at a variant allele frequency of 21.2% with a read depth of 2668 (Fig. 3). Sequencing of DNA extracted from an EDTA blood sample, taken from the patient at the time of surgery, did not reveal any germline PIK3CA variants.

Postoperatively, the patient was enrolled in hand therapy to regain full range of motion and functionality. On the basis of her confirmed PROS, she was prescribed alpelisib to address the areas of the hand that could not be debulked at the time of surgery.

## Discussion

Macrodactyly, characterized by increased soft tissue and/or bone of one or more digits or parts of the body, is a rare congenital condition with an incidence rate of 0.035% among all musculoskeletal defects [6, 7]. Macrodactyly of the hand is even more infrequent as compared with macrodactyly of the foot [8].

Incidence of macrodactyly has been found to be similar across sex and geographical regions. Multiple digit involvement, as seen in this case, is 2.6 times more frequent than single digit involvement. Commonly involved digits include the thumb the index and middle finger. In 79% cases, the affected digit was in the median nerve distribution and was associated with fatty infiltration and enlargement of the median nerve [7, 9]. Thus, the case described above seems to encompass the most frequent picture of hand macrodactyly.

Historically, the pathogenesis of macrodactyly was thought to be related to neurofibromatosis [11, 12]. This was later replaced by the concept of ‘Nerve territory-oriented macrodactyly’ which attributed the excessive and unimpeded digital enlargement to an abnormal nerve supply [13]. However, the true etiology in most cases of isolated macrodactyly remains unknown [6]. Wu *et al*. [7] reported somatic PIK3CA mutations in 10 out of 12 cases of hand macrodactyly included in their study. Activating PIK3CA gene mutations was detected in the affected fat, nerve, and skin tissues of all 10 cases. The mutations detected were exclusively gain of function type, previously reported in PROS.

PROS is an umbrella term, proposed in 2015, that includes a broad and heterogenous group of rare developmental disorders of embryogenesis which range from segmental overgrowth of limbs to megalencephaly capillary malformation polmicrogyria and CLOVES syndromes [14, 15]. Somatic activating variants in PIK3CA, occurring in the post zygotic stage, impart a selective growth advantage to the affected tissue. The phenotypic presentation is determined by the developmental phase at which the mutational event occurred and the subsequent distribution of the mutated cell’s progeny as well as the activating strength of the variant [16]. Thus, the diversity in the clinical presentation, severity, and progression of these disorders is multifactorial.

With growing understanding of PROS, the grouping and classification of this group of disorders continues to evolve. Well-described entities such as CLOVES and MCAP represent one end of the spectrum with patients having most of the manifestations [17]. But at the same time there are individuals who manifest only limited aspects and may not conform to preexisting stereotypes. This has led to the proposal of a dyadic approach to the diagnosis and terminology of this entity [18].

The extensive overlap and divergent manifestations along with the emergence of novel phenotypes necessitate a multidisciplinary approach and use of all appropriate screening tools for deep phenotyping in each case [17]. For instance the case described here seems at the outset to be an isolated, nonprogressive macrodactyly and was treated with debulking surgery. In the absence of the genetic testing ordered by the astute clinical team, the patient would have received a limited workup and follow-up which would have resulted in omission of a more thorough screening to rule out subtler yet serious manifestations of PROS such as underlying vascular malformations.

The identification of the PIK3CA gain of function variant has unlocked therapeutic possibilities for use of mTOR inhibitors of the PI3K-Akt-mTOR pathway in order to provide pharmacological treatment not only in cases with progressive disease, but also in non-progressive cases not amenable to debulking [10].

## Conclusion

This case report highlights the importance of molecular testing and thorough phenotyping to identify PROS, in an attempt to further the knowledge of this esoteric entity with an expanding clinical spectrum.
